# Supplementary material for: A functional subset of CD8+ T cells during chronic exhaustion is defined by SIRPα expression
Source: Nat Commun. 2019 Feb 15;10:794. doi: 10.1038/s41467-019-08637-9 (PMC6377614; doi:10.1038/s41467-019-08637-9)
Supplement: Supplementary file 1 — Supplementary Information [file 41467_2019_8637_MOESM1_ESM.pdf]

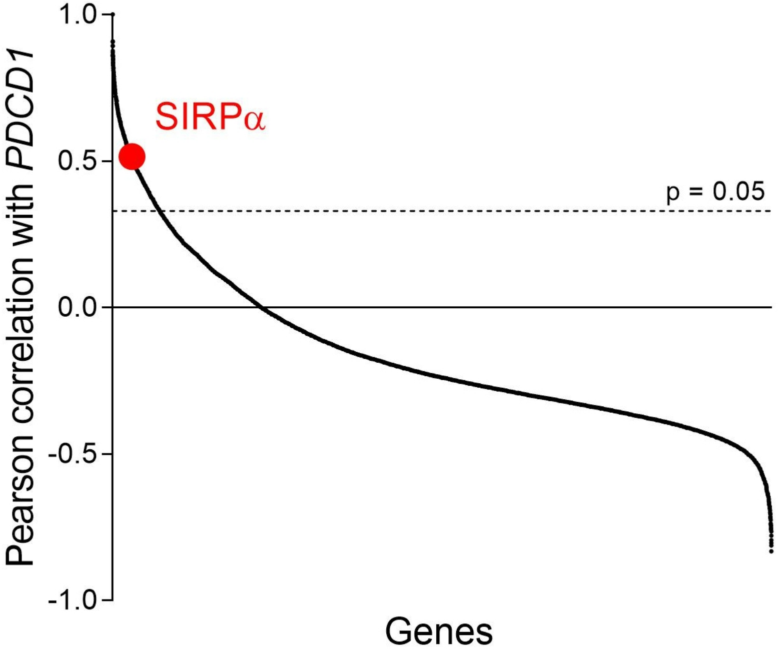

Supplemental Figure 1. Correlation analysis. Sirpα expression significantly correlated with Pdcd1 expression at a Pearson correlation coefficient of 0.516 and p-value of 0.001263. Data from both acute and chronic LCMV infection were used. When all of the genes are organized by order of correlation with the expression pattern of Pdcd1, Sirpα ranked 586 out of 20776 genes (97th percentile). The correlation significance was confirmed for multiple comparisons using the Benjamini-Hochberg procedure ($p<0.05$, $FDR<0.05$).

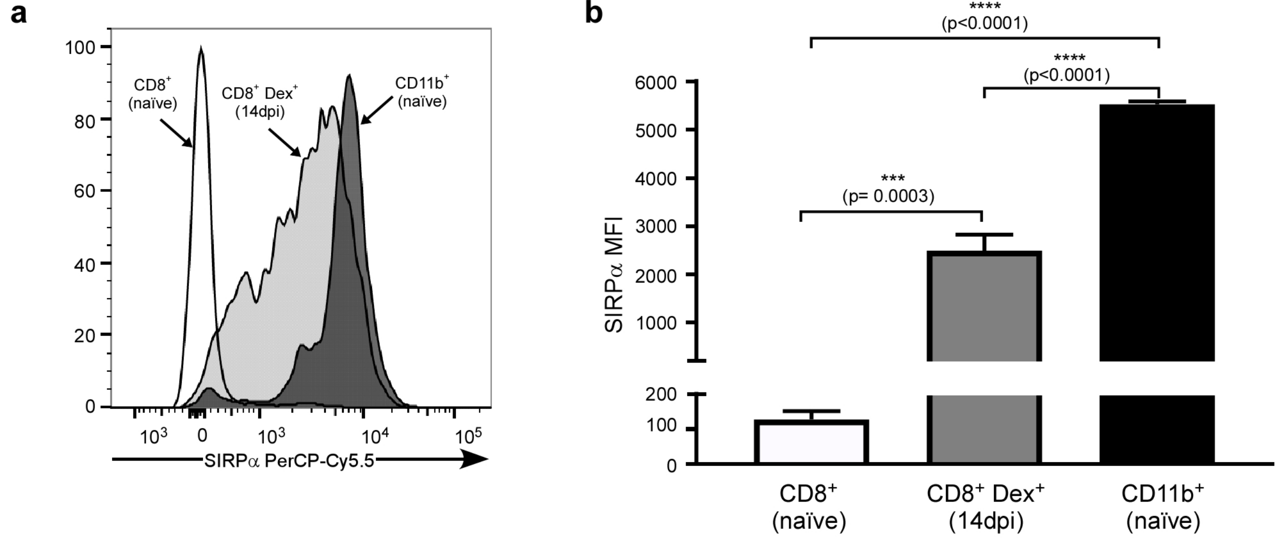

Supplemental Figure 2. SIRPα expression on CD8$^+$ T cells compared to macrophages. Splenocytes from naïve and 14 dpi mice were analyzed by multiparameter flow cytometry for expression of SIRPα. **(a)** A representative histogram overlay is displayed. CD8$^+$ T cells from naïve mice are shown in unfilled solid line, CD8$^+$ dextramer$^+$ T cells from 14 days post-FV infected mice are shown in filled gray and CD11b$^+$ cells (macrophages) from naïve mice are shown in filled black. **(b)** MFI of SIRPα expression where the bar represents the mean, with standard deviation. Data are from one of 3 independent experiments for a total of n=7 naïve and n=10 14 dpi mice. (One-Way ANOVA with Tukey's multiple comparison test).

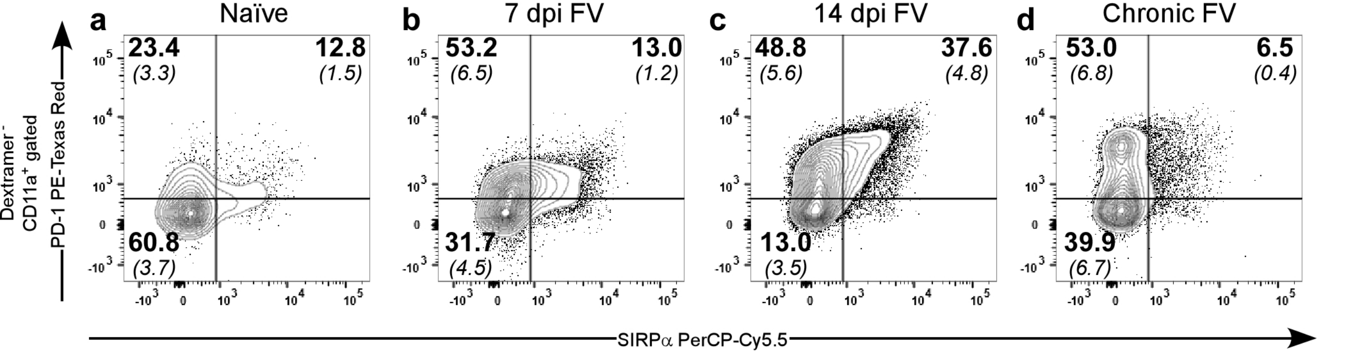

Supplemental Figure 3. PD-1 and SIRPα expression on CD8$^+$ T cells during FV infection. CD8$^+$ splenocytes from **(a)** naïve, **(b)** 7 dpi, **(c)** 14 dpi, or **(d)** chronic Friend virus infected mice were analyzed by flow cytometry for CD11a and FV-D^b gagL dextramer expression, as depicted in Figure 1f-i. The CD8$^+$ dextramer$^-$ CD11a$^+$ were further analyzed by flow cytometry for PD-1 and SIRPα expression during the course of FV infection as shown. The percentage in each quadrant depicts the mean, with standard deviations in parentheses. Numerical data are combined from two independent experiments (n=8 mice at each time-point).

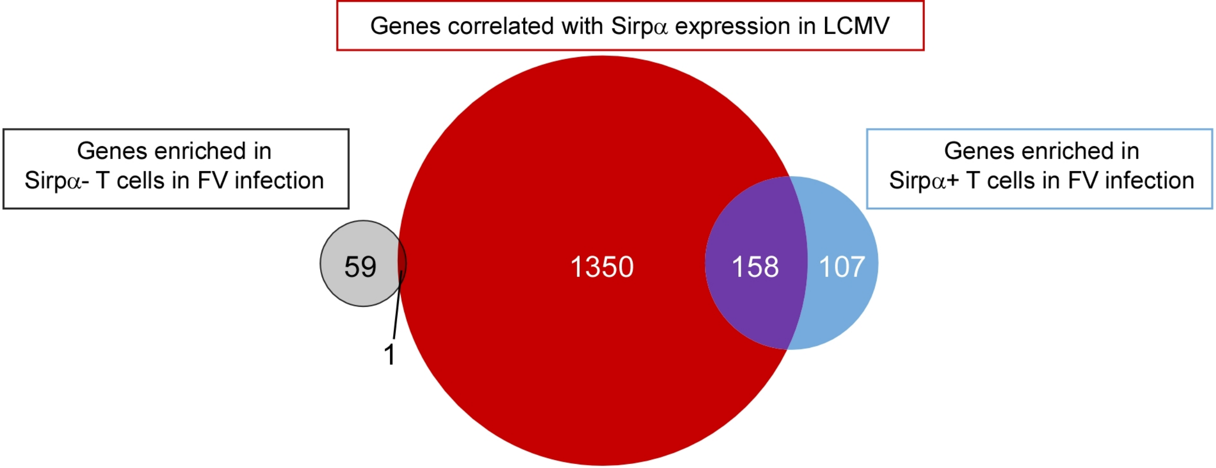

Supplemental Figure 4. Venn diagram comparing gene regulation in FV and LCMV infections. Venn was generated by finding the intersection of three gene lists: 1. genes significantly ($p < 0.05$) correlated with Sirpα expression in T cells during various stages of LCMV infection (Figure 1a) (in red); 2. genes significantly (p-adj < 0.1) enriched in Sirpα- T cells in FV infection (Figure 4) (in gray); and 3. genes significantly (p-adj < 0.1) enriched in Sirpα+ T cells in FV infection (in blue).

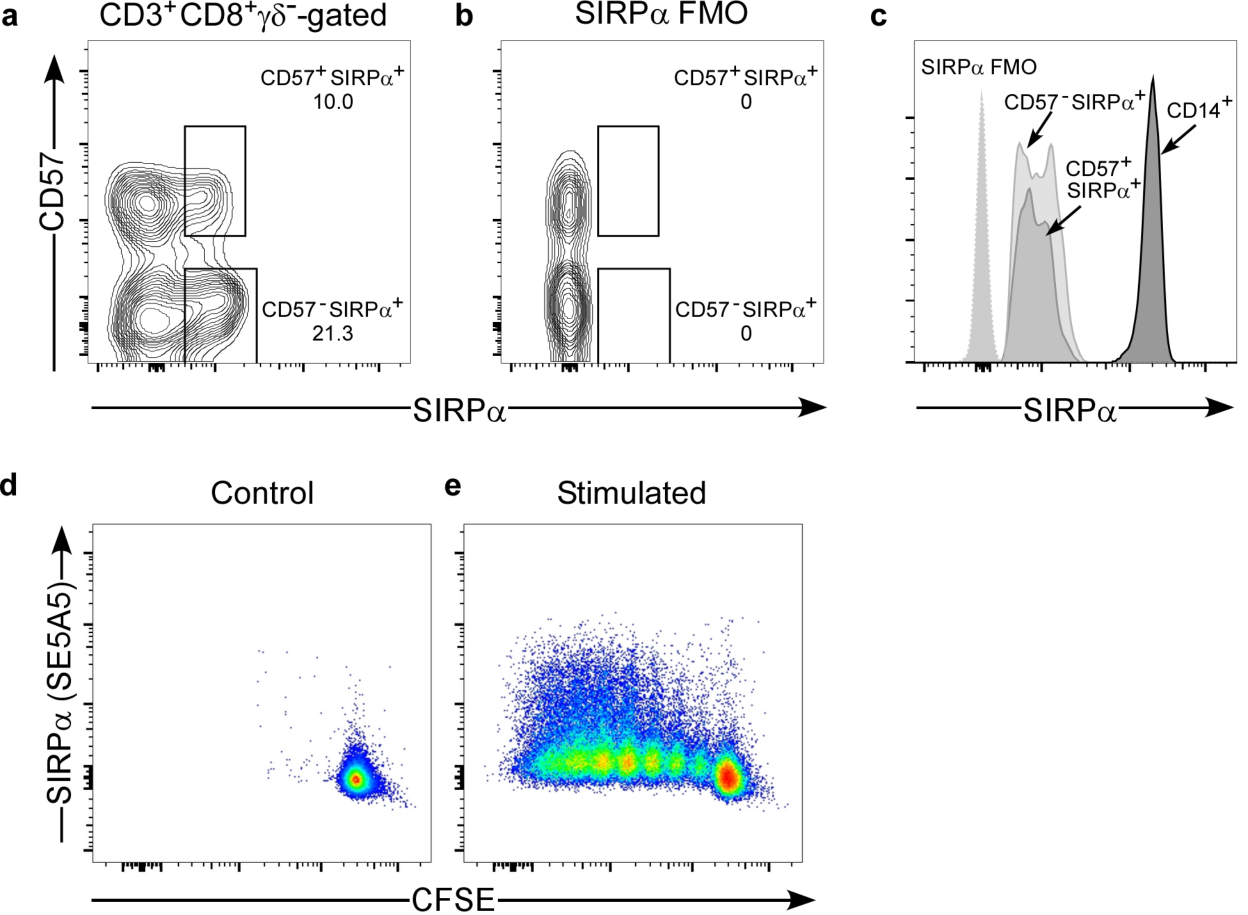

Supplemental Figure 5. HCV-induced SIRPα expression and *in vitro* activation. (a,b) PBMCs from HCV patients were analyzed by flow cytometry for SIRPα expression. $CD3^{+}$ $CD8^{+}$ $CD57^{-}$ $\gamma\delta TCR^{-}$ cells and $CD3^{+}$ $CD8^{+}$ $CD57^{+}$ $\gamma\delta TCR^{-}$ are shown stained for SIRPα in comparison to the full staining panel excluding anti-SIRPα (FMO) control. **(c)** Additionally, the $CD57^{+}$ and $CD57^{-}$ subsets were compared in the histogram to the SIRPα expression levels on $CD14^{+}$ cells from within the same HCV patient's PBMCs. Flow plots are a representative example. **(d,e)** PBMCs were labeled with CFSE and incubated in plates coated with anti-CD3 and anti-CD28 antibodies or control wells. After 5 days *in vitro*, SIRPα expression and CFSE dilution was analyzed by flow cytometry.

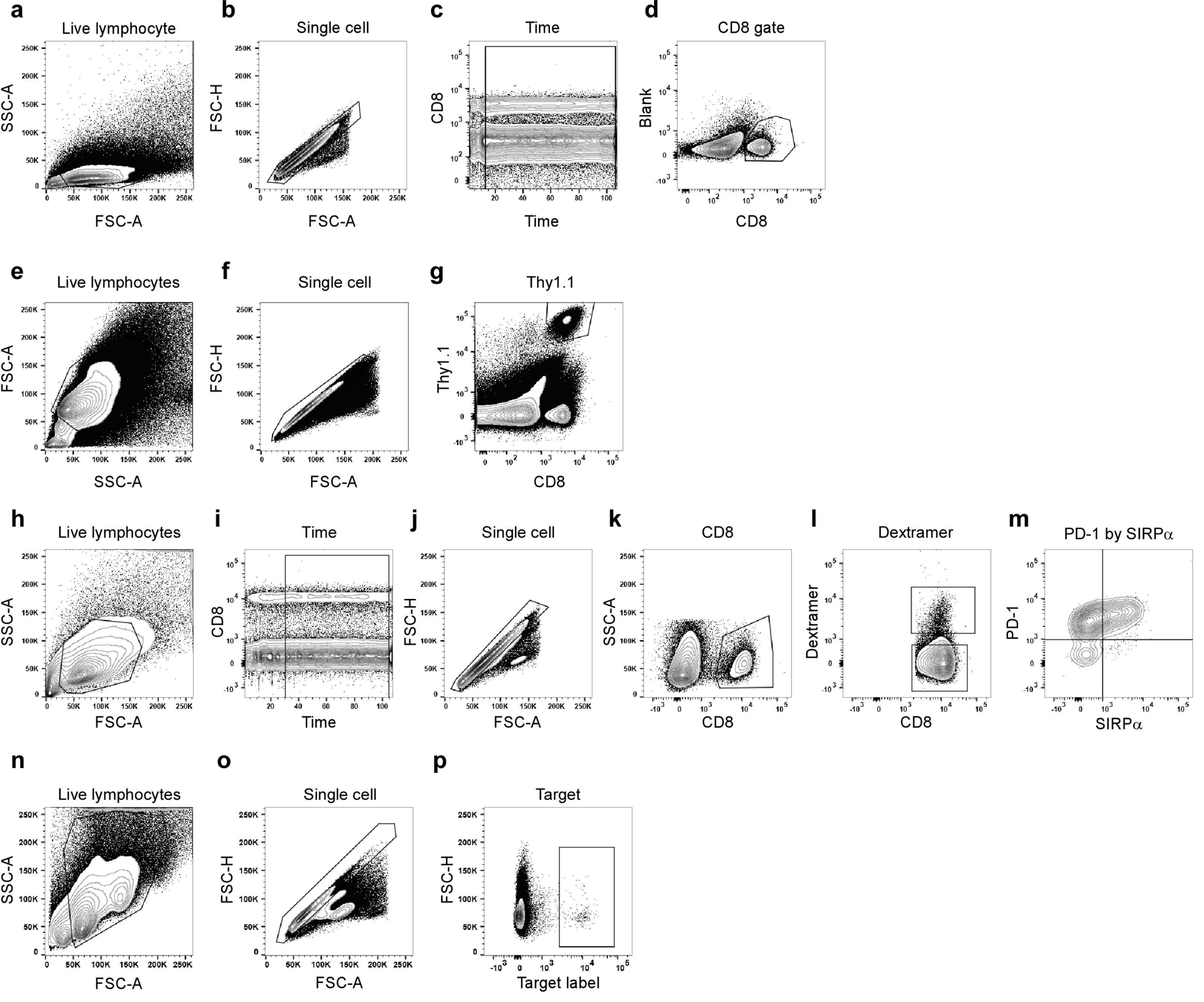

Supplemental Figure 6. Representative gating strategies for FlowJo data analysis. (a-d) The initial gating strategy for Figure 1f-i to identify endogendous FV-specific CD8^{+} T cells expressing PD-1 and SIRPα. **(e-g)** The initial gating strategy for Figure 2 to identify the adoptively transferred FV-specific CD8^{+} Thy1.1^{+} T cells. **(h-m)** The initial gating strategy for Figures 3 and 5 to characterize the phenotype of FV-specific CD8^{+} T cells expressing PD-1 and SIRPα. **(n-p)** The initial gating strategy for Figure 6 to identify the four target cell populations, which were all APC-labelled and then differentially labelled with either two intensities of CellTrace™ Violet or CFSE, as depicted in Figure 6a.

Supplementary Table 1. The top 5 biological processes associated with the SIRPα^{+} subset of CD8^{+} T cells.

	ID	Name	pValue	FDR B&H	FDR B&Y	Bonferroni	Genes from input	Genes in annotation
1	GO:0002684	positive regulation of immune system process	9.134E-12	2.032E-8	1.748E-7	2.787E-8	25	976
2	GO:0051301	cell division	1.332E-11	2.032E-8	1.748E-7	4.064E-8	21	668
3	GO:0002683	negative regulation of immune system process	3.146E-11	3.199E-8	2.752E-7	9.598E-8	17	420
4	GO:0022402	cell cycle process	1.033E-10	7.709E-8	6.630E-7	3.153E-7	28	1385
5	GO:0000278	mitotic cell cycle	1.386E-10	7.709E-8	6.630E-7	4.228E-7	24	1016
