# Supplementary material for: A functional subset of CD8+ T cells during chronic exhaustion is defined by SIRPα expression
Source: Nat Commun. 2019 Feb 15;10:794. doi: 10.1038/s41467-019-08637-9 (PMC6377614; doi:10.1038/s41467-019-08637-9)
Supplement: Supplementary file 3 — Description of Additional Supplementary Files [file 41467_2019_8637_MOESM3_ESM.pdf]

Description of Additional Supplementary Files

File Name: Supplementary Data 1
Description: **Differentially regulated transcripts in SIRPα+ and SIRPα- subsets.** CD8+ T cells from naïve FV-specific Thy1.1+ CD8.TCR mice were transferred i.v. into Y10 mice chronically infected with FV. After 13-15 days, CD8+ cells were purified from the spleens of these recipients using anti-CD8 paramagnetic beads and the Miltenyi MACS systems. Cells were then stained with anti-Thy1.1; anti-CD8; anti-PD-1; anti-SIRPα and sorted into CD8+Thy1.1+PD-1+SIRPα- and CD8+Thy1.1+PD-1+SIRPα+ populations for analysis using a BD FACSAriaIIu. All 325 differentially regulated genes are shown in the table (p-adjusted < 0.1 by Benjamini-Hochberg procedure) and supplementary statistics as outputted by DESeq2 using default settings. baseMean = mean of normalized counts; lfcSE = standard error of log2 fold change; stat = Wald statistic, or the log2 fold change divided by the standard error of log2 fold change; p-value as determined by Wald test; p-adjusted as determined by Benjamini-Hochberg procedure.

File Name: Supplementary Data 2
Description: **SIRPα-associated genes upregulated in LCMV and FV infections.** This table is the list of the genes represented in supplemental figure 4 illustrated as a Venn diagram. The first column lists the genes that were significantly ($p < 0.05$) correlated with Sirpα expression in T cells during various stages of LCMV infection; (2) the genes that are common to columns 1 and 3; (3) genes significantly (p-adj < 0.1) enriched in Sirpα+ T cells in FV infection.
