# Supplementary material for: A functional subset of CD8+ T cells during chronic exhaustion is defined by SIRPα expression
Source: Nat Commun. 2019 Feb 15;10:794. doi: 10.1038/s41467-019-08637-9 (PMC6377614; doi:10.1038/s41467-019-08637-9)
Supplement: Supplementary file 6 — Reporting Summary [file 41467_2019_8637_MOESM6_ESM.pdf]

nature research

Corresponding author(s): *Double-blind peer review submissions: write DBPR and your manuscript number here instead of author names.*

Last updated by author(s): *YYYY-MM-DD*

Reporting Summary

Nature Research wishes to improve the reproducibility of the work that we publish. This form provides structure for consistency and transparency in reporting. For further information on Nature Research policies, see Authors & Referees and the Editorial Policy Checklist.

Statistics

For all statistical analyses, confirm that the following items are present in the figure legend, table legend, main text, or Methods section.

n/a	Confirmed	
☐	☒	The exact sample size (*n*) for each experimental group/condition, given as a discrete number and unit of measurement
☐	☒	A statement on whether measurements were taken from distinct samples or whether the same sample was measured repeatedly
☐	☒	The statistical test(s) used AND whether they are one- or two-sided<br>*Only common tests should be described solely by name; describe more complex techniques in the Methods section.*
☐	☒	A description of all covariates tested
☐	☒	A description of any assumptions or corrections, such as tests of normality and adjustment for multiple comparisons
☐	☒	A full description of the statistical parameters including central tendency (e.g. means) or other basic estimates (e.g. regression coefficient) AND variation (e.g. standard deviation) or associated estimates of uncertainty (e.g. confidence intervals)
☒	☐	For null hypothesis testing, the test statistic (e.g. *F*, *t*, *r*) with confidence intervals, effect sizes, degrees of freedom and *P* value noted<br>*Give P values as exact values whenever suitable.*
☒	☐	For Bayesian analysis, information on the choice of priors and Markov chain Monte Carlo settings
☒	☐	For hierarchical and complex designs, identification of the appropriate level for tests and full reporting of outcomes
☒	☐	Estimates of effect sizes (e.g. Cohen's *d*, Pearson's *r*), indicating how they were calculated

Our web collection on statistics for biologists contains articles on many of the points above.

Software and code

Policy information about availability of computer code

Data collection	B.D. LSRII SORP DIVA version 8.0.1. RNAseq was performed by the Stanford Functional Genomics Facility (Illumina NextSeq). Computing for this project was perfomed on the Stanford Sherlock cluster. Stanford Functional Genomics Facility extracted and generated FASTQ files for each sample, distinguished by the Nextera dual index adapters. Raw reads were trimmed for base call quality (phred >=21) and adapter sequences using Skewer 112. RNAseq was performed by the Stanford Functional Genomics Facility (Illumina NextSeq). Computing for this project was perfomed on the Stanford Sherlock cluster. Stanford Functional Genomics Facility extracted and generated FASTQ files for each sample, distinguished by the Nextera dual index adapters. Raw reads were trimmed for base call quality (phred >=21) and adapter sequences using Skewer 112. Cytof data were collected on the CyTOF2 instrument using CyTOF2 software.
Data analysis	FlowJo software, version 10.2; TreeStar, Inc., Affymetrix arrays from GSE41867 were obtained as CEL files, MAS5 normalized using the “affy” package in Bioconductor, mapped to NCBI Entrez gene identifiers using a custom chip definition file (https:// www.ncbi.nlm.nih.gov/geo/query/acc.cgi?acc=GSE41867) and converted to MGI gene symbols. RNAseq processed reads were aligned to mm10 and read counts were generated using STAR 2.5.3a 113. The R package ‘DESeq2’ was used to normalize read counts, perform differential gene expression analysis, and generate the heat map. Cytof data were de-barcoded and manually analyzed on Cytobank (cytobank.org).

For manuscripts utilizing custom algorithms or software that are central to the research but not yet described in published literature, software must be made available to editors/reviewers. We strongly encourage code deposition in a community repository (e.g. GitHub). See the Nature Research guidelines for submitting code & software for further information.

Data

Policy information about availability of data

All manuscripts must include a data availability statement. This statement should provide the following information, where applicable:

- Accession codes, unique identifiers, or web links for publicly available datasets
- A list of figures that have associated raw data
- A description of any restrictions on data availability

The RNAseq data that support the findings of this study have been deposited in Sequence Read Archive with the project accession code SRP173611. The remaining data that support the findings of this study are available from the corresponding author upon reasonable request. The remaining data that support the findings of this study are available from the corresponding author upon reasonable request.

Field-specific reporting

Please select the one below that is the best fit for your research. If you are not sure, read the appropriate sections before making your selection.

☒ Life sciences ☐ Behavioural & social sciences ☐ Ecological, evolutionary & environmental sciences

For a reference copy of the document with all sections, see nature.com/documents/nr-reporting-summary-flat.pdf

Life sciences study design

All studies must disclose on these points even when the disclosure is negative.

Sample size	Sample sizes were determined by a sample size calculator using a 95% confidence level.
Data exclusions	No data were excluded.
Replication	Experimental replicates are indicated in the figure legends. All replicate experiments were successful.
Randomization	Allocation into experimental groups was not randomized because the experiments were done in age and sex-matched, genetically identical mice.
Blinding	Data identification for experimental animals during collection was done by box number/animal number. Following collection, when numbers were already entered and were unalterable, box/animal number codes were associated with experimental groups so that negative or naive controls could be used to set analysis gates. For Fig. 4, RNAseq was done blinded.

Reporting for specific materials, systems and methods

We require information from authors about some types of materials, experimental systems and methods used in many studies. Here, indicate whether each material, system or method listed is relevant to your study. If you are not sure if a list item applies to your research, read the appropriate section before selecting a response.

Materials & experimental systems

n/a	Involved in the study	
☐	☒	Antibodies
☒	☐	Eukaryotic cell lines
☒	☐	Palaeontology
☐	☒	Animals and other organisms
☐	☒	Human research participants
☒	☐	Clinical data

Methods

n/a	Involved in the study	
☒	☐	ChIP-seq
☐	☒	Flow cytometry
☒	☐	MRI-based neuroimaging

Antibodies

Antibodies used	A700-anti-CD8 (53-6.7, eBioscience 56-0081-82, lot E08952-1633; 1/800) or PacBlue-anti-CD8 (53-6.7, BD Pharmigen 558106, lot 38114; 1/400); FITC-anti-CD11a (2D7, BioLegend 101006, lot B165666; 1/400); PE-CF594-anti-PD-1(J43, BD Horizon 562523, lot 7243896; 1/200); PE-Cy7-anti-Thy1.1 (H1551, eBioscience 25-0900-82, lot 4300740; 1/1000); FITC-anti-CD107a (1D4B, BD Pharmigen 553793, lot 02482; 1/100); PE-anti-Tim3 (8B.2C12, eBioscience 12-8571-81, lot E008713; 1/400); PE-anti-Lag3 (C9B7W, BD Pharmigen 552380, lot 0000054474; 1/50); FITC-anti-Fas (Jo2, BD Pharmigen 15404, lot M045159; 1/100); PE-Cy7-anti-CD43 (1B11, BioLegend 121218, lot B132711; 1/1000); BV605-anti-CD44 (IM7, BD Horizon 563058, lot 7177869; 1/1000); BV711-anti-CD40 (3/23, BD Biosciences 740700, lot 6326576; 1/400); PE-Cy7-anti-CD278 (C398.4A, BioLegend 313520, lot B135805; 1/200); PE-Cy7-anti-CD62L (MEL-14, eBioscience 25-0621-82, lot E07577-943; 1/1000); PE-anti-CD122 (TM-b1, BD Biosciences 553362, lot 24161; 1/200); FITC-anti-KLRG1 (2F1, eBioscience 11-5893-82, lot E09834-484; 1/800); PE-Cy7-anti-CX3CR1 (SA011F11, BioLegend 149016, lot B216575; 1/200); APC-anti-CD47 (miap301, eBioscience 17-0471-82, lot 4301458;

	1/100); PerCP-Cy5.5-anti-SIRPα (P84, BioLegend 144010, lot B252132; 1/100). For FV-specific H-2Db/Abu-Abu-L-Abu-LTVFL staining, APC- or PE-Db gagL-MHC Dextramer (Immudex, Copenhagen, Denmark) was used at 1/25. PE-anti-EOMES (Dan11mag, eBioscience 12-4875-82, lot E10466-1634;1/200); PE-Cy7-anti-Tbet (eBio4B10, eBioscience 25-5825-82, lot 4277988; 1/200); A700-anti-Ki67 (B56, BD Pharmigen 561277, ;lot 7073537; 1/200); PE-anti-TCF-1 (S33-966, BD Biosciences 564217, lot 8081983; 1/200) and APC-anti-human granzyme B (GRB05; Molecular Probes Invitrogen GRB05, lot 1908524; 1/50). (MAb 34), which is specific for F-MuLV glycosylated Gag protein (mAb34 was produced at RML/NIAID/NIH as a culture supernatant). MAb 34 binding was detected with FITC-labeled goat anti-mouse IgG2b (R12-3, BD Pharmigen 553395, lot 32885; 1/800).
Validation	Dendritic cells, macrophages and mononcytes from mice with targeted SIRPα gene disruptions completely lose reactivity with mAb p84 (anti- SIRPα) even though their SIRPβ expression is normal. These results indicate specificity of p84 for SIRPα without cross reactivity for SIRPβ

Animals and other organisms

Policy information about studies involving animals; ARRIVE guidelines recommended for reporting animal research

Laboratory animals	As stated in the methods section: For LCMV studies26, female 4-6 week old C57BL/6J mice from NCI and Thy-1.1+ P14 TCR transgenic mice 108 that recognize the H-2Db gp33 epitope were used where indicated. For Friend virus studies, mice were female (C57BL/10 x A.BY) F1 (Y10) (H-2b/b, Fv1b, Rfv3r/s, Fv2r/s) and FV-specific Thy1.1+ CD8.TCR transgenic mice between 12-24 weeks of age at the beginning of the experiments.
Wild animals	*Provide details on animals observed in or captured in the field; report species, sex and age where possible. Describe how animals were caught and transported and what happened to captive animals after the study (if killed, explain why and describe method; if released, say where and when) OR state that the study did not involve wild animals.*
Field-collected samples	*For laboratory work with field-collected samples, describe all relevant parameters such as housing, maintenance, temperature, photoperiod and end-of-experiment protocol OR state that the study did not involve samples collected from the field.*
Ethics oversight	As stated in the methods section: The use of all animals for LCMV studies was conducted in accordance with and approved by the Yale University IACUC guidelines. For FV studies, Mice were treated in accordance with RML IACUC-approved animal use protocols following the regulations and guidelines of the Animal Care and Use Committee of the Rocky Mountain Laboratories and the National Institute of Health Office of Laboratory Animal Welfare.

Note that full information on the approval of the study protocol must also be provided in the manuscript.

Human research participants

Policy information about studies involving human research participants

Population characteristics	The study included fifteen HCV-infected patients. Ten patients underwent at least one previous treatment with interferon, the other five were treatment naïve. The distribution of variables set as age, sex, history of previous IFN treatment, history of transplantation, HCV genotype, and HCV infection status is detailed in the methods.
Recruitment	Patients with HCV infecttion were asked during their routine visit to Stanford Liver Clinic fif they wanted to participate. The study lasted from November 2013 to May 2016.
Ethics oversight	Study protocol number 13859 approved by the Stanford University Institutional Review Board.

Note that full information on the approval of the study protocol must also be provided in the manuscript.

Flow Cytometry

Plots

Confirm that:

- [x] The axis labels state the marker and fluorochrome used (e.g. CD4-FITC).
- [ ] The axis scales are clearly visible. Include numbers along axes only for bottom left plot of group (a 'group' is an analysis of identical markers).
- [x] All plots are contour plots with outliers or pseudocolor plots.
- [ ] A numerical value for number of cells or percentage (with statistics) is provided.

Methodology

Sample preparation	Splenocytes were isolated by tissue homogenization through a 100-□m filter and RBCs were removed using lysis buffer (0.15 M NH4Cl, 10 mM KHCO3, 0.1 M EDTA)
Instrument	Becton Dickinson LSRII
Software	FlowJo software (version 10.2; TreeStar, Inc.)
Cell population abundance	These target cell and effector cell populations were then placed in a 2 hr in vitro cytotoxic killing assay at a 1:4 target:effector

Cell population abundance	ratios of post-sorted cells were either (10,000:40,000 cells) or 1:10 (10,000:100,000) as indicated in the figure legend. As stated in the methods section, Sorted populations were ≥95% pure in all assays as determined by flow cytometry.
Gating strategy	For flow cytometric analysis, live lymphocytes were gated using a SSC-A and FSC-A gate. Cells were then gated by time to exclude artifact caused by erratic sample flow and by FSC-H and FSC-A to exclude doublets. All specific gating strategies are shown in supplementary figure 6.

☒ Tick this box to confirm that a figure exemplifying the gating strategy is provided in the Supplementary Information.
